# Do Dogs Prefer Helpers in an Infant-Based Social Evaluation Task?

**DOI:** 10.3389/fpsyg.2019.00591

**Published:** 2019-03-29

**Authors:** Katherine McAuliffe, Michael Bogese, Linda W. Chang, Caitlin E. Andrews, Tanya Mayer, Aja Faranda, J. Kiley Hamlin, Laurie R. Santos

**Affiliations:** ^1^Department of Psychology, Boston College, Chestnut Hill, MA, United States; ^2^Department of Psychology, Yale University, New Haven, CT, United States; ^3^Department of Psychology, Harvard University, Cambridge, MA, United States; ^4^Department of Zoology, University of Cambridge, Cambridge, United Kingdom; ^5^Department of Organismic and Evolutionary Biology, Harvard University, Cambridge, MA, United States; ^6^Department of Psychology, University of British Columbia, Vancouver, BC, Canada

**Keywords:** social evaluation, helper, hinderer, infancy, domestic dogs, cooperation

## Abstract

Social evaluative abilities emerge in human infancy, highlighting their importance in shaping our species' early understanding of the social world. Remarkably, infants show social evaluation in relatively abstract contexts: for instance, preferring a wooden shape that helps another shape in a puppet show over a shape that hinders another character (Hamlin et al., 2007). Here we ask whether these abstract social evaluative abilities are shared with other species. Domestic dogs provide an ideal animal species in which to address this question because this species cooperates extensively with conspecifics and humans and may thus benefit from a more general ability to socially evaluate prospective partners. We tested dogs on a social evaluation puppet show task originally used with human infants. Subjects watched a helpful shape aid an agent in achieving its goal and a hinderer shape prevent an agent from achieving its goal. We examined (1) whether dogs showed a preference for the helpful or hinderer shape, (2) whether dogs exhibited longer exploration of the helpful or hinderer shape, and (3) whether dogs were more likely to engage with their handlers during the helper or hinderer events. In contrast to human infants, dogs showed no preference for either the helper or the hinderer, nor were they more likely to engage with their handlers during helper or hinderer events. Dogs did spend more time exploring the hindering shape, perhaps indicating that they were puzzled by the agent's unhelpful behavior. However, this preference was moderated by a preference for one of the two shapes, regardless of role. These findings suggest that, relative to infants, dogs show weak or absent social evaluative abilities when presented with abstract events and point to constraints on dogs' abilities to evaluate others' behavior.

## Introduction

Social evaluation is a core part of the human moral sense: humans tend to prefer helpful individuals and avoid harmful individuals, behaviors which undoubtedly contribute to our ability to work cooperatively in large groups (Hamlin, 2013). Remarkably, some research suggests that social evaluation may be present from infancy. In a first demonstration of early-emerging social evaluation, Hamlin et al. (2007) presented 6- and 10-month-old infants with a puppet show in which an agent (a wooden shape with googly eyes) attempted, but failed, to climb a hill. The agent was either helped or hindered by another shape. In a preference task, infants preferred the helpful shape. These findings were the first to suggest that social evaluative abilities may be present from very early in life, and have now been replicated and extended numerous times (for reviews see Holvoet et al., 2016; Margoni and Surian, 2018; but see also Salvadori et al., 2015 for a failure to observe preferences for prosocial over antisocial agents in 9-month-olds). Overall, this work has led some scholars to argue that capacities for social evaluation may be part of a system of “core” knowledge, which extends to other conceptual domains (Spelke, 2000; see also Hamlin, 2013).

The finding that social evaluation is deeply rooted in ontogeny raises the question of whether it might be similarly deeply rooted in phylogeny. Do other species show signatures of social evaluation or is this ability unique to our species? Research on animals suggests that indeed social evaluation may be important to sustaining productive cooperative^1^ relationships outside of humans. Across a range of taxa, individuals from cooperative species evaluate others based on past behavior and use this evaluation to guide their own decisions (Russell et al., 2008; Subiaul et al., 2008; Herrmann et al., 2013; Abdai and Miklósi, 2016). For instance, reef-dwelling client fish watch cleaner fish (*Labroides dimidiatus*) cleaning other clients and choose to approach those who behaved cooperatively (Bshary and Grutter, 2006). Similarly, chimpanzees (*Pan troglodytes*) recruit collaborators who have behaved cooperatively with others in previous interactions (Melis et al., 2006). More recently, social evaluation in animals has been shown to cut across taxonomic lines. For instance, coral trout (*Plectropomus leopardus)*, a fish species which hunts collaboratively with moray eels, can quickly learn to recruit effective eel collaborators (Vail et al., 2014). Social evaluation has also been shown in more abstract contexts: using an infant-inspired paradigm involving moving shapes, recent work has shown that bottlenose dolphins (*Tursiops* spp) expect agents to interact with helpers (Johnson et al., 2018) while bonobos (*Pan paniscus*) show a reliable preference for hinderers (Krupenye and Hare, 2018).

Building on this work showing that several animals species evaluate conspecifics and other cooperators, a series of recent studies have investigated whether animals socially evaluate humans (Anderson et al., 2012, 2013; Kawai et al., 2014). For example, tufted capuchin monkeys (*Cebus apella*) discriminate between good and bad human partners across two contexts: they preferentially accept food from a human who has previously helped another human (Anderson et al., 2012) and from a human who has previously shown reciprocity toward another human (Anderson et al., 2013). While these results indicate that certain primate species have the ability to socially evaluate humans, they are difficult to reconcile with the natural social ecology of nonhuman primates, as non-human primates would not typically benefit from choosing cooperative *human* partners in the wild.

Domestic dogs (*Canis familiaris*), on the other hand, are dependent on humans for a range of benefits and so present an ecologically valid model for studying animals' evaluations of human actors. Additionally, like humans, dogs show within and between-species cooperation (Miklósi, 2007; Kaminski and Marshall-Pescini, 2014), leading to a range of contexts in which social evaluation may be beneficial. Finally, because pet dogs and human infants grow up in the same environment, attend to human social cues, and witness similar social stimuli in their environment, many theorists have argued that dogs are a particularly useful comparison for shedding light on potentially human-unique social traits more generally (Hare and Tomasello, 2005; Topál et al., 2014; Johnston et al., 2015).

Dogs cooperate with conspecifics and with humans across a range of real and experimental contexts (Miklósi, 2007; Bräuer et al., 2012, 2013; Ostojić and Clayton, 2013; Kaminski and Marshall-Pescini, 2014). Perhaps because of this, dogs attend to several aspects of their human partners' behavior that could indicate their cooperative tendency: for instance, dogs prefer humans who are friendly (Vas et al., 2005), informative (McMahon et al., 2010), reliable (Takaoka et al., 2014), cooperatively communicative (Petter et al., 2009; Pettersson et al., 2011), winners of playful games (Rooney and Bradshaw, 2006) and, at least in some contexts, familiar (Győri et al., 2010). These studies suggest that dogs pay attention to many features of humans, which likely serves them well in their cooperative relationships with human partners.

Building on these paradigms, a suite of recent studies has begun to probe dogs' social evaluative abilities (reviewed in Abdai and Miklósi, 2016), specifically asking whether, like humans, dogs show a preference for helpful over unhelpful individuals. These studies can be categorized broadly in one of two ways. First, some studies investigate first-party evaluation—contexts in which the subject dog has direct experience with a helpful or unhelpful individual (*direct* evaluation). A second category of studies investigates third-party evaluation (also known as ‘social eavesdropping')—contexts in which the subject dog indirectly observes interactions occurring between others (*indirect* evaluation).

Within the category of work on *direct* evaluation, several studies have examined whether dogs can distinguish between helpful and unhelpful humans based on their own interactions with each person. For instance, in Carballo et al. (2015), dogs learned over trials to prefer a “generous” experimenter who would share food with them over a “selfish” experimenter who would eat the food before they could access it. In support of the idea that experience with social interactions is needed to facilitate this discrimination, only adult dogs but not puppies appear to show this effect (Carballo et al., 2017). However, an open possibility is that dogs' performance in these studies can be explained with an alternative explanation: namely that dogs simply come to associate a certain individual (here, the generous individual) with food, and it is this association that drives dogs' preferences. One recent study accounted for this alternative food association interpretation in its design. In this study, Nitzschner et al. (2012) demonstrated that dogs preferred to associate with a “nice” human—someone who behaved affectionately toward them—rather than a “mean” experimenter who ignored them. Thus, although more evidence is surely needed, dogs appear to be able to form impressions of humans with whom they have directly interacted.

Work on dogs' evaluation in *indirect* contexts has generated more mixed findings (see Abdai and Miklósi, 2016). Marshall-Pescini et al. (2011) examined whether dogs socially eavesdrop on others; specifically, they tested whether dogs pay attention to nice and mean individuals when they are not the direct recipients of nice or mean behavior. In their task, dogs watched a human beggar approach a generous human from whom they received a treat and approach a selfish human who deprived them of a treat. In a choice task, dogs showed a preference for the generous over the selfish human, providing evidence that dogs can socially evaluate others in indirect contexts. However, as with the possibility of a food confound in the work described above, further work building on this paradigm suggested that dogs' preference for a “nice” human was instead a preference for a location associated with food (Freidin et al., 2013; Nitzschner et al., 2014). In line with this second interpretation, other work has shown that dogs prefer “sharing” over “non-sharing” actors, even in relatively nonsocial tasks; for instance, in which the recipient of generous or selfish behavior is a box as opposed to a human (Kundey et al., 2011). Taken together, these studies hint that dogs track and use information about who has previously been associated with food sharing, even when they are uninvolved bystanders watching interactions between other agents or objects. However, rather than reflecting a preference for prosocial behaviors, these tendencies may reflect that dogs are simply savvy about how they will most readily obtain food. Indeed, a recent study by Piotti et al. (2017) explored whether dogs are sensitive to helpfulness in a paradigm that controlled for the possibility that dogs prefer those associated with food. The researchers introduced a condition in which a “nice” individual who spoke in a high-pitched voice and established eye contact with the dog was not associated with food (i.e., was not helpful in showing dogs how to access food) and compared this to one in which the “nice” individual was associated with food (i.e., was helpful in showing dogs how to access food). They additionally compared these conditions to two other conditions in which the experimenter ignored the dog (ignoring but helpful, and ignoring and not helpful). They found that dogs did not show a preference for the helpful individual (ignoring niceness), nor did they show a preference for the nice individual (ignoring helpfulness), providing further evidence that dogs' social evaluative abilities in indirect contexts are importantly limited.

To our knowledge, there is only one remaining case of putative evidence for dogs' social evaluative abilities in indirect contexts (Chijiiwa et al., 2015). In this study, dogs watched their owner ask one of two people for help accessing an object in a jar. In one condition, the helper assisted each dog's owner in opening the jar while a neutral agent did nothing. In another condition, the nonhelper refused to assist each dog's owner by turning away following their request while a neutral agent did nothing. Dogs were then given a choice to approach and receive food from either the helper vs. neutral person (in the first condition) or the nonhelper vs. neutral person (in the second condition). Dogs were presented with four trials, which meant that they received food from their chosen agent before trials two, three and four. Dogs showed no preference for the helpful over neutral agent. However, they avoided the nonhelper relative to the neutral agent. These findings suggested that dogs may be able to socially evaluate in indirect contexts, at least when their owner is the target of helpful or unhelpful behavior. Additionally, these results were suggestive of a negativity bias—preferential attention to negative information—a bias exhibited by human infants (Hamlin et al., 2010) and bonobos (Krupenye and Hare, 2018). However, these results must be interpreted with caution because (1) dogs did not show an aversion to the nonhelper on the first trial (see Abdai and Miklósi, 2016) suggesting that the pattern of reinforcement between trials may have influenced their behavior and (2) there were important asymmetries in how negative vs. neutral actions were performed which may also have affected their avoidance of unhelpful agents.

Thus, although results are mixed, there is some evidence that domestic dogs and human infants show similarities in their ability to track helpful and unhelpful individuals, consistent with the possibility that individuals in both species benefit from being able to quickly evaluate prospective social partners. However, based on work conducted to date there is a key difference in the contexts in which social evaluation has been demonstrated in infants and dogs. Specifically, human infants have been shown to engage in social evaluation in relatively *abstract contexts*— infants interact with shapes rather than people— suggesting that social evaluation in infants may be generalizable. By contrast, work on social evaluation in dogs has to date focused only on whether dogs are able to evaluate good and bad *humans*. While human-evaluation tasks are clearly ecologically valid for dogs, they leave open the question of whether dogs share human infants' ability to extract relevant social information from more abstract contexts. Answering this question will shed light on the strength and flexibility of dogs' social evaluative abilities, providing hints about the importance of these abilities for domestic dogs.

Here we address this question by adapting the original human infant paradigm from Hamlin et al. (2007) for use with domestic dogs. Dogs watched a puppet show in which an agent (a red circle with googly eyes) attempted to climb a hill and was either assisted in climbing by a helper or prevented from doing so by a hinderer shape. Previous work suggests that dogs view moving shapes as social beings (Gergely et al., 2013, 2015, 2016), and thus we were hopeful that dogs would see these shapes as social beings in our task. After seeing this puppet show, dogs were then presented with a choice task in which they could approach the helper or hinderer shape. We predicted that, like infants, dogs would show a preference for helpers. Additionally, we examined whether dogs spent more time investigating the helper or hinderer. We reasoned that dogs may spend longer investigating helpers, if they did indeed show a preference for them. However, we also thought it possible that dogs would spend longer investigating hinderers, consistent with existing evidence that negative social information may be particularly salient to humans (Hamlin et al., 2010), bonobos (Krupenye and Hare, 2018) and possibly to dogs (Chijiiwa et al., 2015). Finally, we examined the number of times that dogs engaged with their handlers during presentations of the helping and hindering events. That is, whether or not they looked at their handlers or otherwise attempt to interact with them; for instance, by looking back at, nuzzling, or putting their head on their handler's lap. Looking back at humans is particularly interesting, as several studies have used this behavior as an indicator that dogs are attempting to engage humans in helping them solve a problem (Miklósi et al., 2003). Here we expected dogs to differentiate between helping and hindering events, but did not have a strong prediction about the directionality of this effect. If dogs showed a strong preference for helpers, they may find helping events more engaging and thus engage with their handlers more while watching helping. In contrast, dogs may find hindering events surprising or unsettling and may thus engage more in response to hindering. Our main aim in designing this study was to provide as close a replication to existing infant work as possible, allowing for a valid comparison of the social evaluative abilities of domestic dogs and infants. We also wished to contribute to the existing literature on dogs' social evaluative abilities, which is quite mixed (Abdai and Miklósi, 2016), by testing dogs' evaluation of helpers and hinderers in a non-food context, thereby removing a factor that has complicated interpretations from past designs.

## Methods

### Subjects and Design

We tested 27 dogs (15 females; Mean age = 6.45 years, Standard deviation = 2.81, Range = 1.70–11.87) at the Canine Cognition Center at Yale University. Dogs were of varying breeds (see Table S1 for breed information). Four additional dogs were tested but excluded due to failure to make a choice within the choice interval (3) and because their handler released them before the choice presentation had been completed (1). This study was conducted after piloting different versions of the puppet show to bring the method in line with infant protocols. Two of the dogs in our final sample participated in earlier versions of the study. These subjects were tested with different stimuli and the interval between sessions was nearly 2 years. Our goal was to test as many subjects as possible (with a maximum of 40) in the time that our main experimenter, who had been trained over several months, was available to run the puppet show. Our final sample of 27 is consistent with similar work on infants (Hamlin et al., 2007 tested 28 6- and 10-month old infants in Experiment 1).

We employed a within-subject design. All dogs were presented with four events (two helping events and two hindering events). Events were presented in alternating order and starting event was roughly counterbalanced across subjects (16 dogs saw the helper event first).

### Set-Up

**Figure 1:**
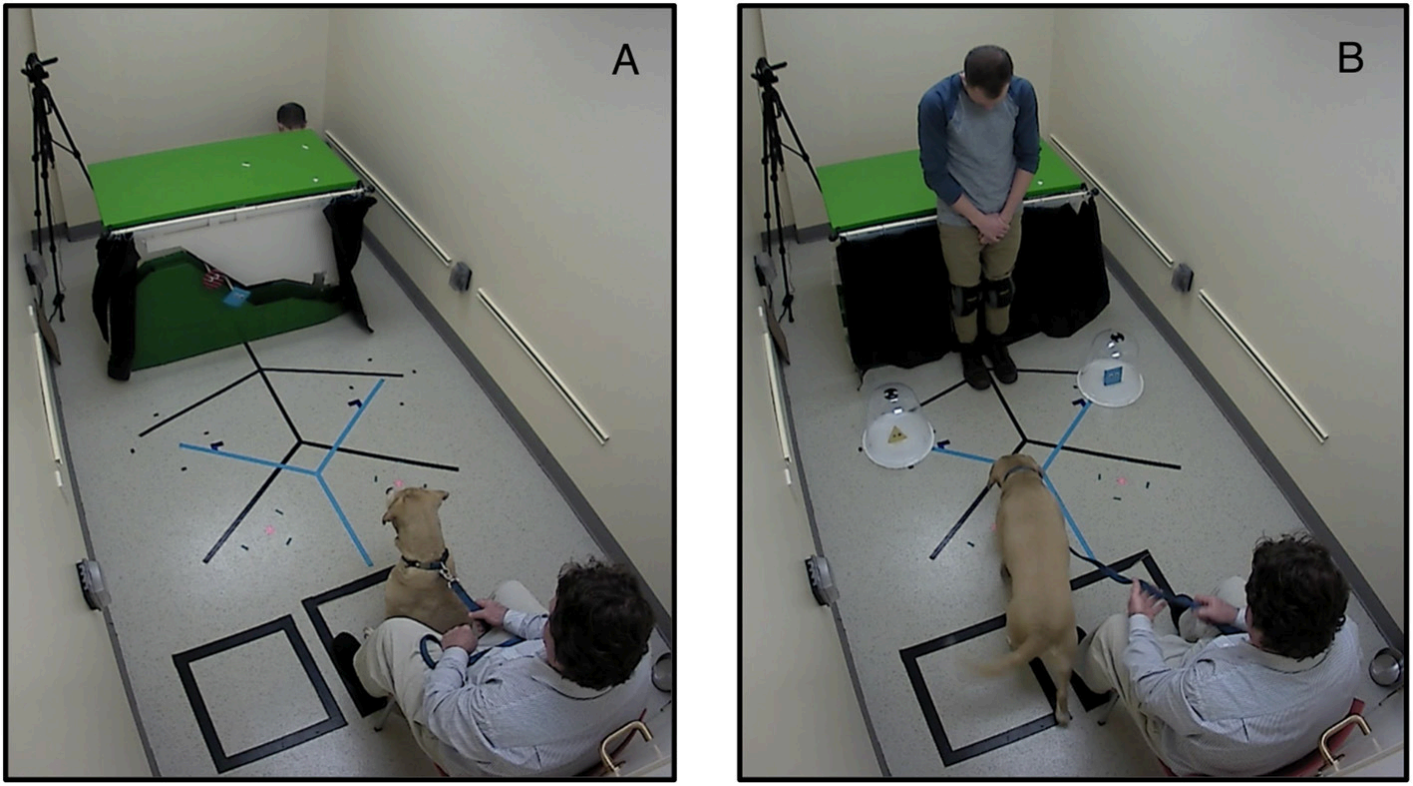
Testing set-up showing a participating dog **(A)** watching the puppet show and **(B)** choosing between the helper and hinderer shapes. Written informed consent was obtained from the depicted individuals for the publication of these images.

Dogs were tested in a small room (6.5 × 12.5 feet; Figure 1), accompanied by their guardians who handled them throughout the experimental session. Guardians sat in a chair with their back to the door and were instructed to position the dog roughly in the middle of their legs. To assist with positioning, a black rectangle was marked on the floor with black tape (Figure 1). A video camera was placed behind the puppet show stage, which recorded the dog and the guardian. Additionally, a ceiling-mounted camera captured a birds-eye view of experimental sessions as depicted in Figure 1. See S2 for a more detailed diagram of room measurements.

The puppet show stage was created using a small table, foamcore, duct tape, and a black shower curtain hung from a rod. The shapes were made of foamcore and wood so that they would make a noise when brought into contact (see Figure S1 for photograph of shapes). Each shape was covered with duct tape for coloration. We chose to use blue and yellow colors for two reasons. First, because these were the colors used in Hamlin et al. (2007). Second, because previous research has shown that dogs can tell them apart (Neitz et al., 1989; Jacobs et al., 1993; see Figure S4 for shapes from a dog's eye view; Pongrácz et al., 2017). Stripes were placed on the target agent in order to align it with the upward angle on the hill and to further differentiate the agent from the helpful and hindering shapes. The agent's eyes were glued to gaze toward the top of the hill to emphasize its goal of climbing upwards, known to be critical for infant social evaluation (Hamlin, 2015).

### Procedure

Our procedure was modeled after Hamlin et al. (2007). Dogs watched a puppet show depicting a “helper” shape assisting an agent achieve its goal of climbing a hill and a “hinderer” shape preventing an agent from achieving its goal of climbing a hill (Figure 1 and Video S1).

Before watching the puppet show, dogs were brought into the testing room and given a few moments to acclimatize to the room. However, they were prevented from exploring the puppet show stage and the area behind it. During this period, the experimenters explained the task to the dog's handler and asked them to keep their eyes closed for the duration of the puppet show. This was done so that handlers would not know which shape was the helper and which was the hinderer, thus preventing cueing during the choice period. Additionally, handlers were asked to try their best to keep dogs positioned roughly in the middle of their legs and oriented toward the show.

### The Puppet Show

To conduct the puppet show, an experimenter crouched under the table, behind the stage (Figure 1) and controlled the shapes using short wooden dowels (see Figure S3 for detailed diagram and measurements of hill). When the dog was in position, the experimenter opened the curtains, at which point the dog saw the agent resting at the bottom of the hill. A squeak sound was made using a rubber squeaker and the agent was moved slightly (rotated to and fro) to attract the dog's attention. The dog then saw the agent attempting, but failing, to climb the hill. They saw two complete attempts and failures. On the third attempt, either the helper or hinderer appeared, depending on event type. In *Helper* events, the second shape (either a yellow triangle or a blue square) appeared at the bottom of the hill and pushed the red circle up the hill, allowing the agent to achieve its goal. In *Hinderer* events, the second shape appeared at the top of the hill and aggressively (with exaggerated, forceful motions) pushed the agent down the hill, preventing it from achieving its goal. At the end of the scene, the agent remained still for 10 s to allow the dog to look. After this period, the curtain was closed and the next scene was initiated with another squeak and shape “wiggle” to orient the dog.

During the show, timing was controlled so that each attempted ascent by the red square was approximately 2 s long, with a rapid 1-s descent over the same distance to simulate “falling” down the hill. The puppet would “rest” for 1 s before attempting to climb the hill again. The agent's climb speed was inverse to its position on the hill; as the agent climbed, the speed of its ascent would slow. In each *helping* and *hindering* event, the second shape would push the red circle twice, moving slightly backwards after the first push to show effort. The agent would pause when not in contact with the helper or hinderer between interactions to emphasize their role in pushing or aiding the agent on the hill. After each event, the agent would pause at the base or top of the hill.

Dogs saw four events in total: two helping and two hindering events. The helper and hinderer shapes were roughly counterbalanced across subjects (blue square was helper for *N* = 16 subjects, yellow triangle was helper for *N* = 11 subjects). Shape was not perfectly counterbalanced because of exclusions and because our original counterbalancing was created for a maximum sample of *N* = 40 dogs.

### Choice Measure

During the two helping and two hindering events, the second experimenter was turned around at the back of the room and was blind to the identity of the helper and hinder shapes. After the dog had seen the puppet show, the second experimenter approached the center of the room, called the dog's name to capture their attention and slowly (and simultaneously) placed the two shapes equidistant from the dog. The shapes were presented in clear plastic domes so that dogs could not mouth them. The domes were positioned exactly 27.25 inches from each other and each dome was placed 35 inches from the dog. To ensure consistency in placement across sessions, the dome positions were marked on the floor in black tape (see Figure 1; see Figure S2 for a detailed measurements of the choice area). After placement, the second experimenter backed away and gave the handler a cue to release the dog. If the dog did not look at both domes, the second experimenter tapped the domes to attract the dog's attention. By tapping on the shapes, either with equal force or with slightly more force on one or the other (the one the dog had not seen), we tried to make sure that the dog saw both shapes before moving on to the next part of the procedure. The second experimenter then called the dog's name to center their attention, backed away and gave the handler a cue to release the dog. Handlers were invited to open their eyes for the choice phase of the task. We counterbalanced whether the helper or hinderer was presented on the right or left.

Dogs had a period of 30 s to make a choice and to explore the two shapes. Choices were coded when the dog had one or both paws in or on the choice area, which was demarcated with black tape for ease of live and video coding. After 30 s, the session was terminated. The dog was then led out of the testing room and the handler was debriefed.

### Coding and Analysis

Choice data— specifically, whether the dog chose the helper or hinderer— were live coded by the second experimenter who was blind to which shape was the helper or hinderer. In addition to a live coder, we recruited a second coder to watch and code video-recorded sessions. Sessions were coded from the birds-eye view videos that were captured by a ceiling-mounted camera. However, videos from the camera inside the room were referred to if visual access was occluded in the birds-eye view videos. Our video coder watched all sessions for (1) dog attention; (2) experimenter error; (3) handler error. Attention varied across events, but all included subjects saw at least one of the helping and one of the hindering events. Within events, we ensured that they either oriented toward the stage during the “squeak” or when the shapes first appeared. During the choice phase, all included dogs either oriented toward the second experimenter or had a chance to see both shapes before making a choice. Our video coder also recorded any instances of handler engagement by the dogs. Engagement was coded when dogs turned their heads to look at their handler or otherwise interacted with them (e.g., nuzzling). After watching videos to code for these variables, our video coder re-watched all videos and coded for shape exploration time. Dogs were considered to be exploring the shapes when they were in close proximity to one of the domed shapes. This criterion was met when the dog was directed toward and touching or close to touching (within a few inches of) the dome or base. Pawing was counted within the exploration time. Please see Table S3 for a summary our dependent measures of interest.

Following video coding, we recruited a third coder to serve as a reliability coder. Our reliability coder coded videos of the choice procedure, which included only the presentation of shapes and the dog's choice (i.e., she did not watch the demonstration phase and was thus blind to condition). Thus, for almost all sessions, we had four sources of data for dog choices: live coding from our “live coder,” video coding and exploration coding from our “video coder” and reliability coding from our “reliability coder.” In our only exception, live coder failed to note down the dogs' choice on the live coding sheet and thus we only had three sources of data for choice coding. In our sample of 27 dogs, there were only four cases of disagreement across these sources of data. In these cases, we relied on consensus across coding sources (e.g., if three sources reported a “square” choice and one reported a “triangle” choice, we recorded “square”). Please see Table S4 for a summary of our choice data sources.

Analyses were conducted in R version 3.3.2 (R Core Team, 2016). We examined three dependent measures of interest. First, we examined whether dogs were more likely to choose the helper or hinderer using two-tailed binomial tests, which compared dogs' choices of the helper shape to 50% probability of choosing the helper due to chance. Second, we tested whether dogs spent longer exploring the helper or hinderer shape. To test this, we first employed a two-tailed paired t-test. We tested for normality by examining a quantile-quantile plot, which displays the correlation between our sample distribution (differences) and the normal distribution. Because the majority of our points fell along the 45° reference line, we considered our data to meet the assumptions of a paired *t*-test. Second, we ran a linear mixed model which allowed us to examine interactions of interest while controlling for repeated exploration measures within dog. Subject identity was fit as a random intercept in our mixed model. Third, we tested whether dogs showed more handler engagement during Helper or Hinderer events using generalized linear mixed models (GLMMs) with a logit link function with the presence or absence of engagement (yes = 1, no = 0) as our dependent measure. Again, we included subject identity as a random intercept to control for repeated measures within subject. For both our linear and generalized linear models, we assessed the importance of predictors by including them in a full model and comparing the model with a predictor of interest to one without the term of interest. Model comparisons were conducted using Likelihood Ratio Tests (LRTs) using the command “drop1.” Models were fit using package “lme4” (Bates et al., 2012, 2015). Across all our analyses, we additionally explored whether dogs showed a consistent side bias (e.g., a preference for the object presented on the right) and whether they preferred one of the shapes over the other (i.e., a preference for the yellow triangle or the blue square).

## Results

### Choice: Did Dogs Preferentially Approach Helpers?

**Figure 2:**
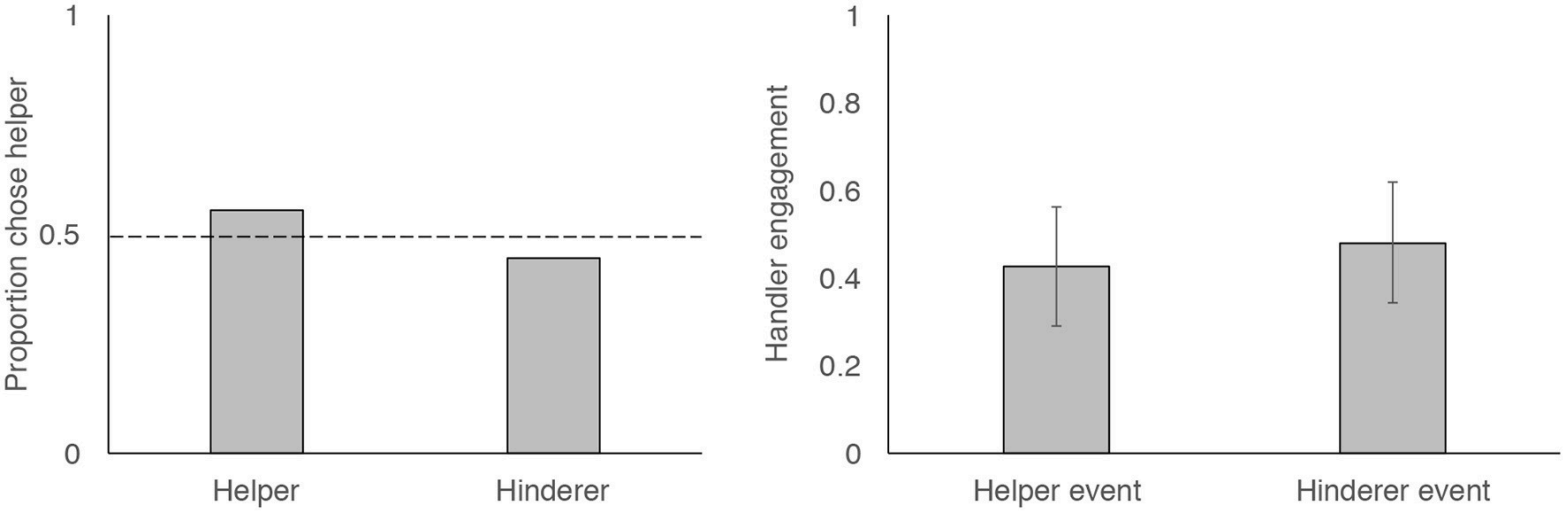
Figures showing proportion of dogs who chose to first approach the helper vs. the hinderer **(left)** and probability that dogs engaged with handlers during Helper and Hinderer events **(right)**. Dotted line shows expectation of chance-level behavior and error bars show 95% confidence intervals.

Fifteen of our 27 dogs chose to approach the helper shape first, which did not differ from chance (Figure 2; two-tailed binomial test, *p* = 0.701). Dogs were no more likely to approach the square than the triangle (10 approached the square first, binomial test, *p* = 0.248). Choice data thus suggest that dogs did not show a preference for the helper shape, nor did they show a preference for the square or the triangle. However, we did see a significant preference for shapes presented on the dogs' left side: 20 of the 27 dogs approached the shape that was presented on the dog's left side (binomial test, *p* = 0.019).

### Exploration: Did Dogs Preferentially Explore Helpers?

To examine whether dogs spent more time exploring the helper or hinderer shape, we tallied the total amount of time dogs spent in proximity to one shape or the other and compared them with a two-tailed paired *t*-test. We found that dogs spent more time exploring the hinderer than the helper (*t* = −2.27, df = 26, *p* = 0.032). There was no difference in dogs' exploration of the shape placed on the right or the left (*p* = 0.4). However, dogs spent longer exploring the triangle than the square (*t* = −3.5, df = 26, *p* = 0.002).

**Figure 3:**
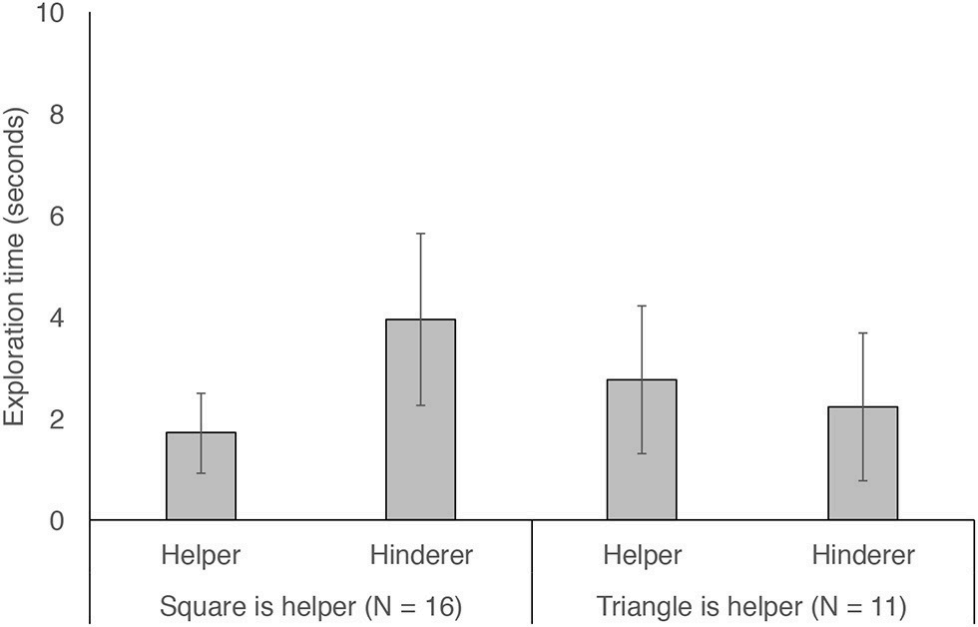
Time spent exploring the helper and hinderer shapes in cases in which the helper was the blue square (left two bars) or the yellow triangle (right two bars). Error bars show 95% confidence intervals.

To understand whether dogs' preferential exploration of the hinderer could be explained by their preference for the triangle, we conducted a general linear mixed model with exploration time fit as a function of role (helper or hinderer) and shape (was the helper shape the square or triangle) and the interaction between these two terms. We found that the interaction between role and shape was significant (LRT, $X_{1}^{2}$ = 9.55, *p* = 0.002). As Figure 3 shows, this interaction was due to the fact that dogs showed greater exploration of the hinderer in cases in which the triangle was the hinderer (i.e., in which the square was the helper). This same exploratory preference was not seen in cases in which the triangle was the helper, although we had fewer of these cases due to the sampling imbalance mentioned above. To test whether side influenced dogs' exploration, we reran our model with side (the side on which the helper was presented: left or right) entered as a control variable. Including this term did not change our results (LRT, $X_{1}^{2}$ = 9.55, *p* = 0.002), nor did its inclusion improve model fit (LRT, $X_{1}^{2}$ = 0.22, *p* = 0.64). Model output from all models can be found in Table S2.

### Handler Engagement: Were Dogs More Likely to Engage Their Handlers During Helping Events?

Dogs often engaged with their handlers during the event presentations (Figure 1). However, they were no more likely to engage during the Helper or Hinderer events. Our GLMM showed that event type (helper vs. hinderer) was not a significant predictor of dog's probability of handler engagement (*p* = 0.477). We additionally examined whether engagement became more common across time by including event number (1–4) as a predictor and examining the interaction between event number and event type (Helper vs. Hinderer). However, these effects were not significant predictors of dogs' engagement behavior (*p*s > 0.5). To test whether side influenced dogs' engagement, we reran our reduced model (event as a predictor) with side (the side on which the helper was presented: left or right) entered as a control variable. Including this term did not change our results (*p* = 0.5), nor did its inclusion improve model fit (LRT, $X_{1}^{2}$ = 0.43, *p* = 0.51). Model output from all models can be found in Table S2.

## Discussion

To our knowledge, our study is the first to adapt a well-established infant social evaluation paradigm (Hamlin et al., 2007) to test domestic dogs. Reasoning that the ability to evaluate helpfulness would be beneficial to domestic dogs due to their reliance on humans, we predicted that, like human infants, domestic dogs would show a preference for helpers over hinderers. However, across three measures we found no strong support for this prediction: dogs were no more likely to approach the helper shape than the hinderer shape, no more likely to engage handlers during the helpful events than during the hindering events, and no more likely to explore hindering individuals independently of the individuals' color and/or shape. On this last point: dogs in our study did show greater exploration of the hindering individual on our exploration measure, indicating that they may have found hindering behavior to be more puzzling and/or interesting than helping behavior, thus warranting extra investigation. However, this effect must be interpreted with caution, as dogs' exploration of hindering shapes was moderated by a preference for the triangle shape over the square shape.

Our choice measure was most analogous to the preference measure used in past infant work and suggested that dogs had no preference for the helper over the hinderer. This is intriguing given that past infant work has shown a strong and early-emerging preference for helpers in this paradigm: In Hamlin et al. (2007) study, a large majority of 6- and 10-month old infants (26 of 28 infants tested in Experiment 1) chose to interact with the helpful as opposed to the hindering shape, and follow-up replications (Hamlin, 2015) showed a similar rate of helper preferences (but see Scarf et al., 2012; Hamlin, 2015 for evidence that infants do not prefer helpers in certain circumstances, and Salvadori et al., for a replication failure in a different context). These data are interesting in light of other work which shows that dogs *avoid* unhelpful individuals in indirect contexts in a ‘live action' paradigm, in which they witness human agents interacting (Chijiiwa et al., 2015). Specifically, dogs avoid people who refuse to help their owner. Taken together with our findings, these results suggest that dogs' social evaluative abilities may be restricted to more ecologically-valid contexts. By contrast, infants have a more general ability to extract relevant social information from a wider range of contexts.

While our choice data did not reveal a preference for helpers, it did reveal a significant side bias: dogs were more likely to choose to approach the domed shaped that had been placed on the left side of the room. We are not entirely sure why this happened. One possibility is that there was more equipment stored on the left side of the room (the camera was placed there) so it is possible that the left side of the room was more attractive because it contained more visual stimuli than the right side of the room. A second possibility is that dogs preferred to approach the hinderer's side of the room (recall that the hinderer always emerged from the left side and disappeared into the left side of the hill). A third possibility is that the dogs were avoiding the side of the room where the second experimenter had been waiting with the choice objects. Because we could not counterbalance the side of the recording equipment or the side from which the second experimenter approached due to constraints of our room set-up, we cannot distinguish between these possible explanations for the observed side bias. However, these are merely speculative suggestions, as we had no a priori reason to think that one side of the room would be more attractive to dogs than the other.

Our handler engagement results suggest that dogs frequently engaged their handlers by socially referencing them or using other behaviors. However, they were no more likely to engage during the Helper or Hinderer events. While this is by no means a perfect measure of dogs' responses to these events, we thought handler engagement would provide insight into whether dogs were more interested in or unsettled by one event than the other. For instance, dogs look back at humans when confronted with an unsolvable task (Miklósi et al., 2003) and there is recent evidence that dogs show social referencing toward humans when they encounter a potentially scary object (Merola et al., 2012a,b). Despite this, we observed no differential handler engagement during helper and hinderer events in our task.

Relative to our choice and handler engagement measures, our exploration time measure yielded some intriguing differences in dogs' behavior toward the helper and hinderer shapes. We found that dogs spent longer investigating the hinderer than the helper during the 30 s exploratory period. Preferential exploration of the hinderer is consistent with the idea that dogs did, in fact, distinguish between the helper and hinderer and were perhaps driven to preferentially investigate it out of surprise at its behavior, keeping in mind that this preference was also moderated by the shape/color of the object. This result is in line with previous work showing that in a “live action” paradigm, dogs did not distinguish between a human who helped their owner (the helper) versus one who did nothing (neutral agent), but avoided a human who refused to help their owner relative to the neutral agent (Chijiiwa et al., 2015, but see Abdai and Miklósi, 2016). Taken together with our shape exploration finding, these results suggest that dogs may pay particular attention to unhelpful individuals, avoiding them in some contexts and exploring them in others (our paradigm). Indeed, dogs may show something akin to the negativity bias that has been seen in young infants (Hamlin et al., 2010; see Abdai and Miklósi, 2016 for a review).

While our finding that dogs showed preferential exploration of the hinderer is intriguing, we must be cautious in interpreting it richly for two reasons. First, it is possible that dogs were more likely to explore the hinderer due to activity differences that existed between the helper and hinderer events. One difference is that the hinderer may have contacted the red circle with slightly more force than did the helper in the experimenter's effort to convey hindering behavior. This may have even resulted in a slightly louder sound from the dog's perspective during the hindering events, which could have led to difference in how attention-grabbing the different scenes are. In addition to this possibility, there may have been other differences across the scenes which led to differential attention and thus to differential exploration (e.g., maybe dogs viewed hindering events as more playful than helping events). While these possibilities should certainly be considered, it is also important to note that we kept our events as close to the infant paradigm as possible, thereby making it worthwhile to discuss differences between our dog findings and the existing infant findings. A second reason why we must be cautious in interpreting our exploration result is because we additionally found that dogs spent longer investigating the triangle than the square. Our follow-up model suggested that dogs' exploration time was predicted by an interaction between shape role (helper versus hinderer) and shape (triangle versus square). Thus, what initially appeared to be preferential exploration of the hinderer is likely to be—at least in part—accounted for by a preference for the triangle. Because the triangle was always yellow, this could also be explained by preference for yellow objects. This preference appeared to be particularly pronounced when the triangle was the hinderer, suggesting that dogs were especially drawn to the triangle when it was playing the hinderer role. While these data are suggestive of a potentially interesting additive effect of dogs' interest in hinderers and triangles, it is difficult to make a strong case for this interpretation because we had a slight sampling bias toward the triangle playing the hindering role due to exclusions and our sample size being lower than our planned maximum target. Future work investigating dogs' abstract social evaluative abilities could also include guardian questionnaires which assess whether dogs have more triangle- or square-shaped toys at home (or yellow- or blue-colored toys) and could pre-test dogs for a baseline color and/or shape preference.

Our aim in designing this study was to adapt a method that has been successfully employed in work on social evaluation in young infants. While we believe that we achieved this aim, our close reliance on the infant method resulted in several possible limitations of our study. First, at a high level, our aim to standardize methodology meant that our paradigm was not particularly socially valid for dogs. Future work could adapt the puppet show to use objects and events that would be more familiar to dogs. Second, and in this same vein, it is possible that domestic dogs do not ascribe agency to wooden shapes with googly eyes in the same way that human infants do. While this is certainly a possibility, it is worth noting that previous work has shown that dogs do view moving objects as social interaction partners (Gergely et al., 2013, 2015, 2016). Nevertheless, a difference in agency ascription could explain the discrepancy between our results and past work that has used a live action paradigm (Chijiiwa et al., 2015). However, another possibility is that dogs understood the actions as helping and hindering but this understanding did not result in a preference since they were unaffected by the agents' actions. Had we tested dogs in a second-party context, one in which they were reliant on one of the agents for help, we may have seen a preference for the helper. Future work could explore this possibility. To further probe dog's understanding of helping vs. hindering, future work could also test dogs in a looking-time paradigm (West and Young, 2002; Racca et al., 2009; Marshall-Pescini et al., 2014). A looking time task would provide insight into whether dogs expect the recipient of help/harm to prefer one agent over the other (Kuhlmeier et al., 2003; Hamlin et al., 2007), even if they do not prefer the helpful over the hindering individual.

A final caveat we would like to note is that we have compared our results to those of Chijiiwa et al. (2015) throughout the discussion because, to our knowledge, their study represents the only remaining putative evidence for dogs' social evaluative abilities in indirect contexts. However, we want to emphasize again that the findings from this study must be interpreted with caution (Abdai and Miklósi, 2016). Additionally, it is important to note that other studies have investigated dogs' indirect evaluative abilities using live action paradigms, and have not found evidence that dogs prefer nice and/or helpful people (Nitzschner et al., 2014; Piotti et al., 2017). Thus, before claiming that dogs can more easily evaluate unhelpfulness in a live-action paradigm than in an abstract paradigm, it is important to understand how robust the effects of indirect social evaluation are in human evaluation tasks. We view this as an important next step for future work in this area.

In sum, our study is the first to adapt a well-established infant social evaluation paradigm for use with dogs. We were interested in exploring whether dogs, like infants, can extract relevant social information from relatively abstract events. However, across our three measures, dogs did not show behavior consistent with this ability. These findings add to the ongoing debate about dogs' social evaluative abilities based on direct and indirect experience and complement existing work suggesting that dogs avoid unhelpful humans in a third-party context. Broadly, these findings suggest that while dogs may attend to and use social information about *human* interaction partners in some contexts, these abilities may not generalize to more abstract contexts as they do in infants.

## Ethics Statement

This study was carried out in accordance with the recommendations of Protocol # 2017-11448 which was approved by the Yale Institutional Animal Care and Use Committee. Owners of animal subjects gave written and informed consent before participation in the study.

## Author Contributions

KM, MB, LC, CA, TM, AF, JKH, and LS cotributed to the design of the study and refined the methodology. Analyses were conducted by KM and LC. The paper was written by KM, MB, LC, CA, JKH, and LS.

### Conflict of Interest Statement

The authors declare that the research was conducted in the absence of any commercial or financial relationships that could be construed as a potential conflict of interest.

## Footnotes

^1^For the purpose of this paper, we use the West et al. (2007) definition of cooperation: “a behavior which provides a benefit to another individual (recipient), and which is selected for because of its beneficial effect on the recipient.”

## Supplementary Material

Video 1 Video clip of helping, hindering and choice events.
