# Supplementary material for: Do Dogs Prefer Helpers in an Infant-Based Social Evaluation Task?
Source: Front Psychol. 2019 Mar 29;10:591. doi: 10.3389/fpsyg.2019.00591 (PMC6449837; doi:10.3389/fpsyg.2019.00591)
Supplement: Supplementary file 2 [file Data_Sheet_1.PDF]

Supplementary materials for *Do dogs prefer helpers in an infant-based social evaluation task?*

TABLE OF CONTENTS

TABLE S1. **2**
Breed, sex and age information for dog participants in this study.

FIG. S1. **3**
Photograph of the three shapes and the hill used in Helper and Hinderer presentations.

FIG. S2. **4**
Diagram of the testing room layout, showing precise measurements.

FIG. S3. **5**
Diagram of foamcore hill, showing precise measurements.

FIG. S4. **6**
Dogs' eye view of stimuli.

TABLE. S2. **7**
Model output.

TABLE S3. **8**
Summary of our dependent measures of interest.

TABLE S4. **9**
Summary of our data sources.

Table S1.
Breed, sex and age information for dog participants in this study.

Breed	Sex	Age
Australian Shepherd	F	5
Beagle, German Shorthair Pointer Mix	F	4
Goldendoodle	F	6
Golden Retriever, Bernese Mountain Dog, Chow Chow Mix[1]	M	5
West Highland Whiter Terrier, Poodle Mix	F	4
King Charles Cavalier Spaniel[1]	F	8
Labrador Retriever, Beagle, Pit Bull Mix	F	10
Labrador Retriever, Great Dane Mix	M	3
Golden Retriever	M	10
Australian Cattle Dog, Hound Mix	F	8
Labrador Retriever, Weimaraner Mix	F	6
Pembroke Welsh Corgi	F	10
Pembroke Welsh Corgi	M	9
Goldendoodle	F	7
Dachshund	M	9
Potcake Mix	F	3
Pit Bull, Labrador Retriever Mix	M	5
Jack Russell Terrier Mix	M	7
King Charles Cavalier Spaniel	M	3
Labrador Retriever	F	6
Labrador Retriever, Boxer Mix	F	12
Pit Bull	M	2
Terrier Mix	M	8
Border Terrier Mix	M	3
Golden Retriever	F	12
Skye Terrier, Poodle Mix	M	4
Brittany Spaniel	F	4

[1] These dogs participated in a pilot version of this study which involved different stimuli. The time between their pilot participation and participation in this study was nearly two years.

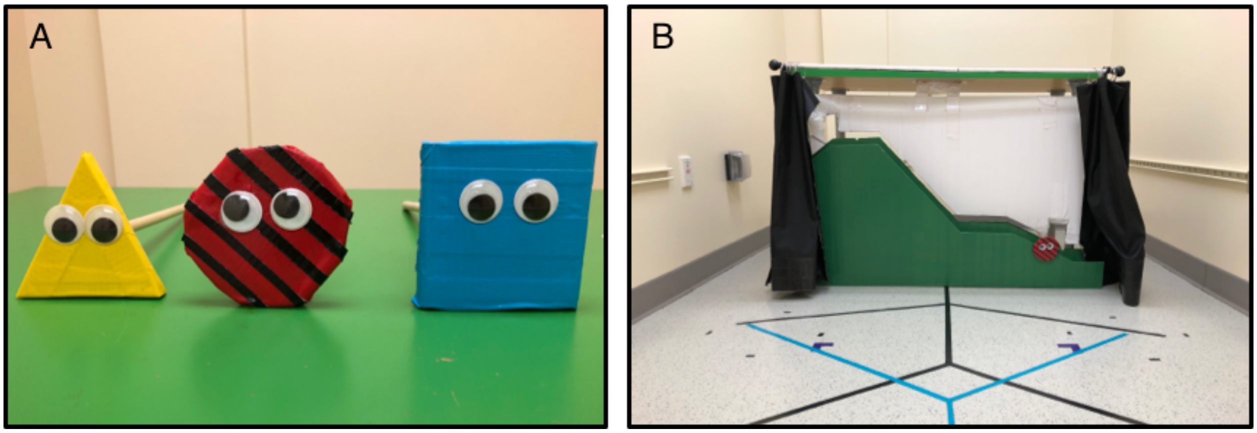

Fig. S1.
Photograph of the three shapes (A) and the hill (B) used in Helper and Hinderer presentations.

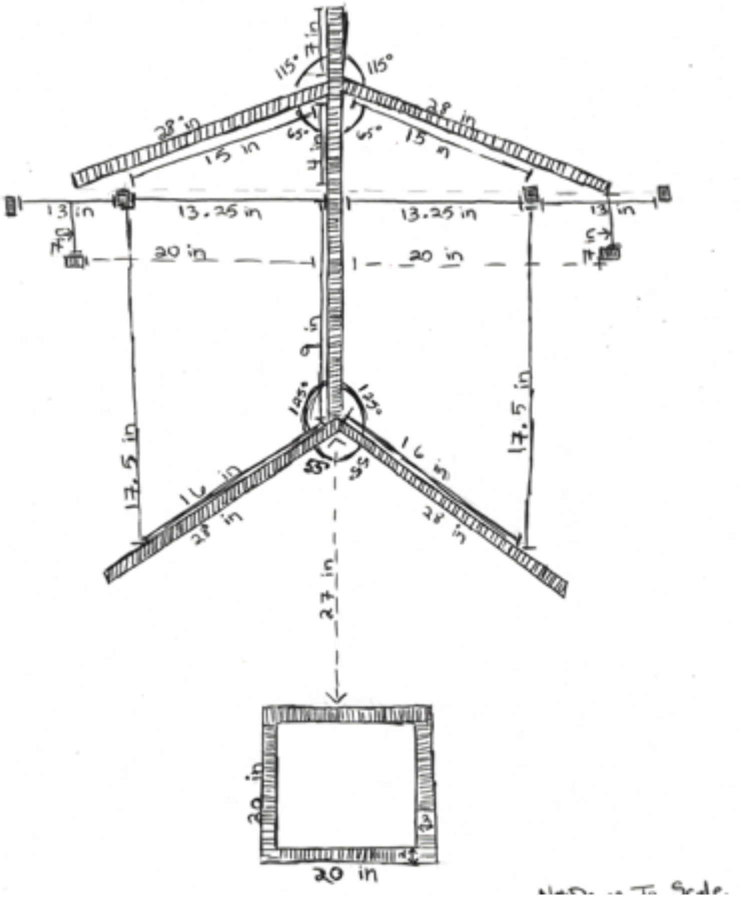

Fig. S2.
Diagram of the testing room layout, showing precise measurements.

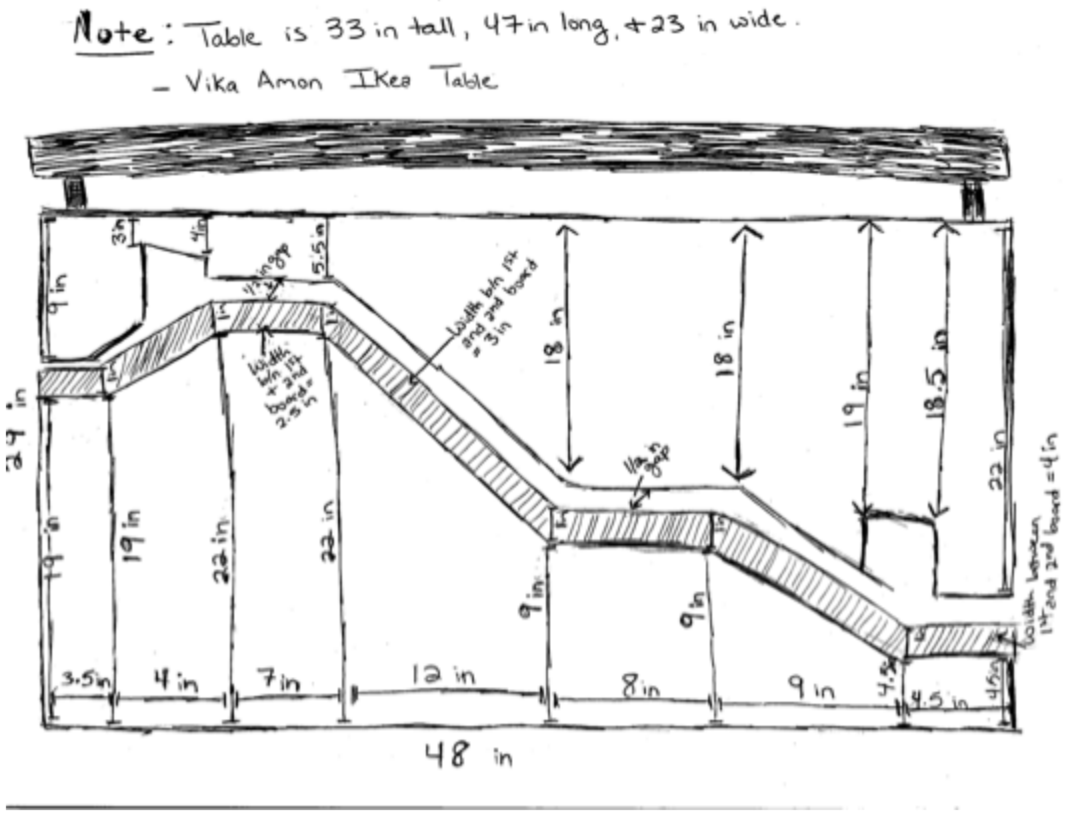

Fig. S3.
Diagram of table and foamcore hill, showing precise measurements.

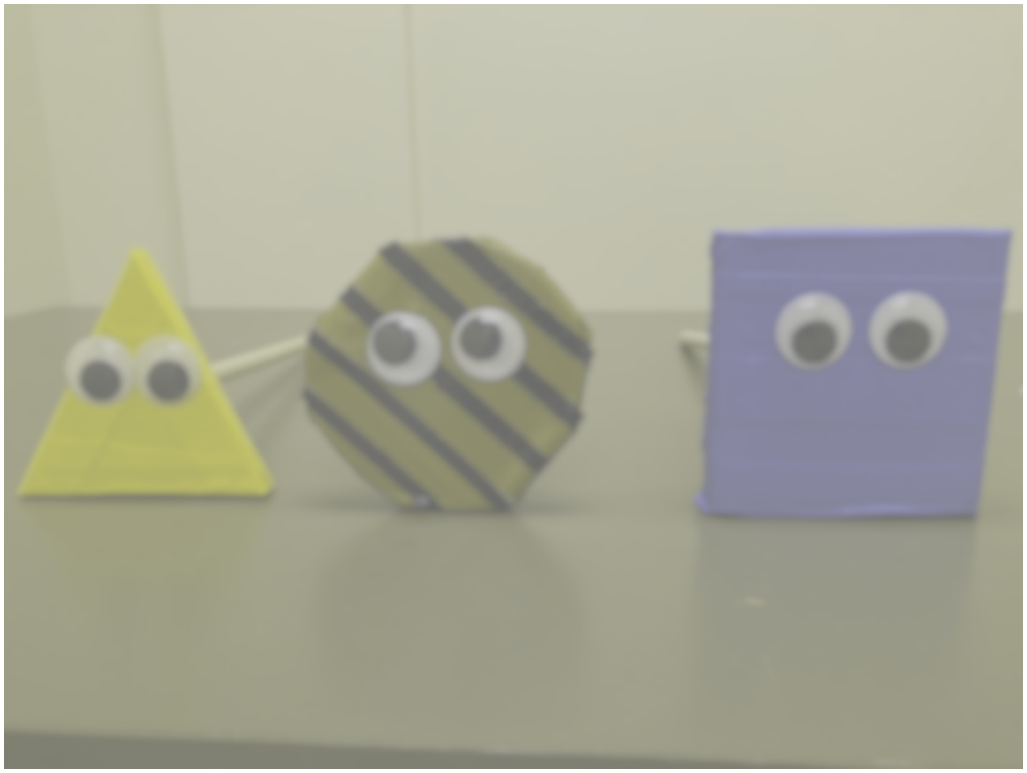

Fig. S4.
Image of our stimuli put through the https://dog-vision.com image processor to simulate a dog's eye view.

Table S2.
Estimate and standard error (s.e.) of effects in mixed models predicting dogs' exploration and handler engagement. Baselines for factors were: Role = Helper, Shape = Square, Side = Left. Table also shows goodness-of-fit statistics

	**Exploration 1**	**Exploration 2**	**Engagement 1**	**Engagement 2**	**Engagement 3**
Intercept	1.72^{**}	1.90^{**}	-0.23	-0.46	-0.19
	(0.59)	(0.72)	(0.86)	(0.46)	(0.60)
Role: Hinderer	2.24^{***}	2.24^{***}			
	(0.54)	(0.54)			
Shape: Triangle	1.05	1.07			
	(0.92)	(0.94)			
Role x Shape	-2.78^{**}	-2.78^{**}			
	(0.85)	(0.85)			
Side of Helper: Right		-0.36			-0.52
		(0.82)			(0.78)
Event: Hinderer			0.52	0.34	0.34
			(1.20)	(0.46)	(0.46)
Act Number			-0.10		
			(0.31)		
Event x Act Number			-0.06		
			(0.44)		
AIC	239.94	240.32	145.25	141.63	143.20
BIC	251.88	254.24	158.66	149.68	153.93
Log Likelihood	-113.97	-113.16	-67.62	-67.82	-67.60
Num. obs.	54	54	108	108	108
Num. groups: Dogs	27	27	27	27	27
Var: Dogs (Intercept)	3.18	3.32	2.64	2.60	2.51
Var: Residual	2.37	2.37			

$^{***}p < 0.001$, $^{**}p < 0.01$, $^{*}p < 0.05$

Table S3.
Summary of our dependent measures of interest.

Dependent measure	Description
Choice	Choices were coded when the dog had one or both paws in or on the choice area, which was demarcated with black tape for ease of live and video coding.
Exploration	Dogs were considered to be exploring the shapes when they were in close proximity to one of the domed shapes. This criterion was met when the dog was directed towards and touching or close to touching (within a few inches of) the dome or base. Pawing was counted within the exploration time.
Engagement	Engagement was coded when dogs turned their heads to look at their handler or otherwise interacted with them (e.g., nuzzling).

Table S4
Summary of our choice data sources[1].

Live Coder	Video Coder		Reliability coder
Choice from observation	Choice from behavioral coding	Choice from exploration coding	Choice from behavioral coding
Y	X	Y	X

[1]An experimenter live coded dogs' choices and recorded these on paper data sheets which were then entered into excel. An independent video coder coded the videos twice: once for behavioral codes and once for exploration time. Finally, a reliability coder coded all final decisions. We then compared data from our video coder and our reliability coder (X entries in the table). There were four disagreements. These disagreements were resolved by examining the codes from the live coder and from the exploration coding (Y entries in the table). Final decisions were determined by consensus across all four cells (XX and YY in the table).
